# Correlation between microbial characteristics and reproductive status of the yak uterus based on macrogenomic analysis

**DOI:** 10.1186/s12917-023-03845-4

**Published:** 2024-01-03

**Authors:** Rui Wang, Meng Wang, Qiaoying Zeng, Libin Wang, Qian Zhang, Sisi Pu, Xin Ma, Jinglei Wang, Yangyang Pan

**Affiliations:** 1 College of Veterinary Medicine, Gansu Agricultural University, Lanzhou, Gansu China; 2 Technology and Research Center of Gansu Province for Embryonic Engineering of Bovine and Sheep & Goat, Lanzhou, Gansu China

**Keywords:** Microbial characteristics, Uterus, Yak, Estrous cycle, Reproductive health

## Abstract

**Introduction:**

This study aimed to investigate the microbial characteristics of yak uteri collected using intrauterine cotton swabs (CS) during different reproductive stages and the correlation of these microbial characteristics with reproductive status.

**Methods:**

We used a macrogenomic approach to analyze the functional aspects of different microorganisms in samples collected during the pre-estrus, estrus, late estrus, and diestrus stages.

**Results:**

The results revealed the presence of 1293 microbial genera and 3401 microbial species in the uteri of yaks at different reproductive stages. The dominant bacterial species varied across the different periods, with *Micrococcus* and *Proteus* being dominant during pre-estrus; *Pseudomonas*, *Clostridium*, *Flavobacterium*, *Bacillus*, and *Staphylococcus* during estrus; *Acinetobacter*, *Bacillus* and *Proteus* during late estrus; and *Pseudomonas*, *Escherichia coli*, and *Proteus* during diestrus.

**Discussion:**

The primary functions of these bacteria are enriched in various metabolic pathways, including carbohydrate and amino acid metabolism, intracellular transport and secretion, post-translational protein modification, and drug resistance. These findings suggest that the microbial diversity in the uterus of yaks plays a crucial role in reproductive regulation and can help prevent reproductive tract-related diseases.

**Supplementary Information:**

The online version contains supplementary material available at 10.1186/s12917-023-03845-4.

## Introduction

Metagenomic techniques have become increasingly popular for studying microbial diversity in various parts of the body, including the gastrointestinal tract and reproductive system. For instance, Jiang et al. [1] utilized metagenomic data to analyze the gastrointestinal microbiota of seven ruminants and found that vitamin B and K2 biosynthesis was more abundant in the gastric microbiota. On the other hand, thiamine, niacin, and pyridoxine biosynthesis were more prevalent in the colon. WGS metagenomic data was utilized by Mariano et al. [2] for a comprehensive characterization of the ruminal ecosystem of cows. The data revealed that *Bacteroidetes* and *Firmicutes* were the two major phyla, while *Proteobacteria* and *Actinobacteria* were highly represented in third place. *Prevotella* and *Bacteroides* were the most abundant genera. Similarly, Virendra et al. [3] used *16S rRNA* sequencing to perform metagenomic analyses of uterine samples and discovered significant differences in the uterine microbiota between healthy mares and those with endometritis. In non-pathogenic states, the uterine and vaginal environments harbor numerous microbial communities, with the genus *Lactobacillus* being the dominant bacterial group in the reproductive tract [4]. However, various factors (such as age, hormones, nutrients, PH, and the environment) can lead to changes in microbial communities [4–6]. For instance, during infancy, the vaginal microbiome is a combination of aerobic and anaerobic bacterial communities consisting of *Prevotella*, *Enterobacteria*, *Streptococcus*, and *Staphylococcus* species [6]. After puberty, the increase of glycogen and decrease in pH due to the estrogenic environment led to the dominance of *Lactobacilli* species. *Lactobacilli* play a crucial role in maintaining the stability of the internal environment by producing lactic acid and bacteriocins, which suppress microorganisms associated with ecological imbalances and reduce the risk of reproductive diseases [7]. Thus, changes in uterine microbiota can affect embryo implantation and pregnancy outcomes [8]. Furthermore, alterations in the vaginal microbiota can interfere with fertilization, implantation, and subsequent embryonic development, leading to fertility treatment failure and a decrease in live birth rate (LBR) [9]. Therefore, studying microbiota diversity in the animal uterine environment is important for reproductive regulation and maintenance of uterine health.

Reproductive hormones play an essential role in the growth and development of animals; their expression in the uterus varies with the estrous cycle. For example, the expression of follicle-stimulating hormone receptor (FSHR) in yaks reaches its peak during estrus, is significantly decreased during late estrus, has lowest expression during the diestrus period, and increases again during the pre-estrus period [10]. The amounts of estrone (E1) and estradiol (E2) released from the uterine muscle layer in female pigs change with variations in luteinizing hormone (LH) and follicle-stimulating hormone (FSH). This is especially significant in female pigs during estrus, where LH and FSH are important regulators of estrogen release from the uterine muscle layer during luteus lysis [11]. These findings confirm the existence of differences in uterine physiological regulation during different estrous cycles. Secretion of estrogen, FSH, and LH peaks during estrus; estrogen, LH, and FSH levels begin to decline during late estrus. However, it has not been reported whether these reproductive hormones cause changes in the uterine microbiota.

Quereda et al. [12] conducted metagenomic analysis of vaginal swabs obtained from Indian cows at different stages. They found that progesterone affects bacterial diversity, and that bacterial diversity during estrus significantly differs from that during the other three estrous cycles. Therefore, reproductive hormones may also cause changes in the uterine microbiota. Diaz-Martínez et al. [13] compared vaginal and uterine microbiota of 48 women who underwent in vitro fertilization (IVF) and found that the relative abundance and diversity of the reproductive tract microbiota in women with repeated implantation failure (RIF) were significantly different from those without RIF. Their study indicated that microbial changes might lead to reproductive diseases. However, collecting live samples from animal uteri is difficult and may introduce pathogenic bacteria, causing endometrial infection and even loss of reproductive ability in cows [14]. Therefore, studying the diversity of the uterine microbiota during the animal estrous cycle may provide a basis for revealing microbial involvement in reproductive regulation.

**Fig. 1:**
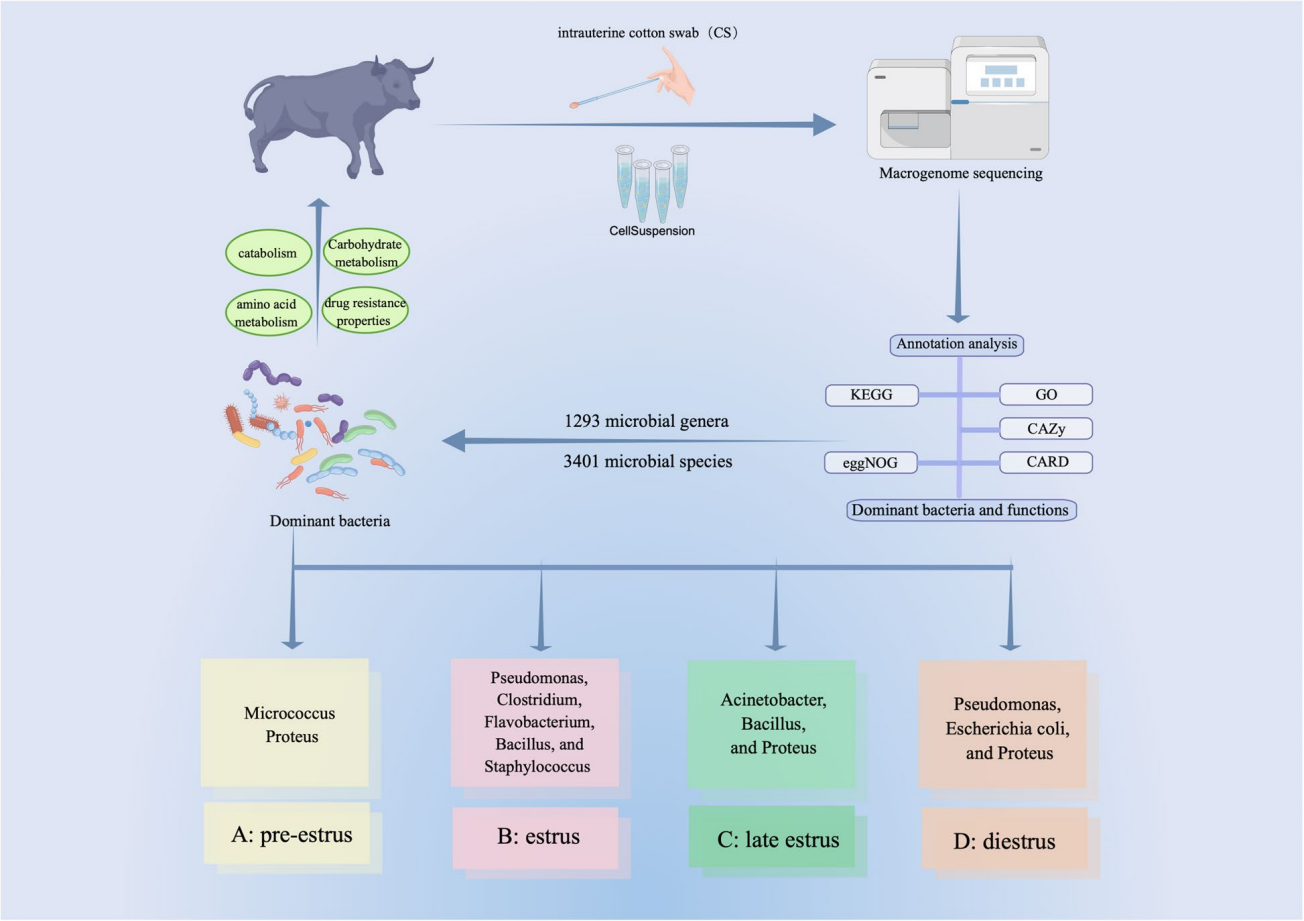
Flow chart for this study by Figdraw

Yaks are unique species found exclusively on the Tibetan Plateau and are known for their remarkable ability to withstand extreme cold, hypoxic, and low-temperature environments. Previous studies have reported significant differences in the physiological state of the yak uterus during different estrous cycles. For instance, the expression of HIF-1α protein in the ovary and fallopian tube was higher in the luteal phase than in the follicular and pregnancy phases. However, in the uterus, the expression of HIF-1α protein is higher in pregnancy than in the follicular and luteal phases [15]. Additionally, Bcl-2 and Bax are dynamically expressed in the placenta of pregnant and postpartum yaks. And They are crucial for the development of the yak fetus and placenta [16]. Yak FSHR is localized in various cells, including surface and glandular epithelial, stromal, smooth muscle, and vascular endothelial cells. The expression of the FSHR protein peaks during the estrus period, significantly decreases during the diestrus period, and increases again during the proestrus period before estrus [10]. Female yaks had a negative energy balance after parturition. This led to an excessive mobilization of fatty acids, which in turn blocked the synthesis and secretion of oestrogen. As a result, reproductive hormones remained at low levels in the late perinatal period, which was the main reason for the delayed recovery of reproductive function after parturition [17]. Despite these findings, little is known about the diversity of the uterine microbiota in yaks. Therefore, this study utilized metagenomic methods to analyze the characteristics of the uterine microbiota during different estrous cycles in yaks, exploring the dominant bacterial groups and the main enriched functional pathways (Fig. 1). These findings provide a reference for revealing the mechanisms of microbial diversity in reproductive regulation and a new method for diagnosing uterine diseases.

## Materials and methods

### Materials

#### Determination of estrus timing

The interval between the onset of estrus in yaks from the previous cycle to the subsequent process was defined as one estrous cycle, generally 18–23 days with an average of 21 days. Changes in estrogen levels cause yaks to undergo regular ovulation, exhibiting different characteristic behaviors before and after ovulation to prepare for subsequent fertilization. This experiment determined the estrus period by directly observing changes in the external genitalia of yaks, rectal palpation to check follicle size, and a comprehensive analysis of estrogen levels.

During the pre-estrus stage, eggs and follicles mature in preparation for ovulation. Palpation examination reveals soft follicles with increased elasticity and distinct fluctuations, thin walls, and flat ovulation fossae. In the estrus stage, eggs are near maturity, and follicular rupture initiates ovulation. On palpation, the follicle wall feels tense, elasticity fades away, walls become unusually thin, and there is a cracking sensation on touching. In the late estrus stage, once ovulation has occurred, the size of the ovary significantly decreases, causing the formation of the corpus luteum where the follicle ruptured. Upon palpation, the corpus luteum is spherical and slightly rigid. In the diestrus stage, once ovulation has occurred, the size of the ovary significantly decreases, causing the formation of the corpus luteum where the follicle ruptured. During this time, the cow’s ovary goes into a quiescent state with no additional follicles developing and no functional corpus luteum. The ovary’s surface becomes smooth to the touch, and the ovarian fossa is deep and obvious.

#### Sample collection

Samples were collected from the Xiahe County Ranch in the Gannan Tibetan Autonomous Prefecture. Three healthy female yaks aged 4–6 years were selected for each of the four estrus stages: proestrus, estrus, metestrus, and diestrus. Samples were collected using the intrauterine cotton swab (CS) method [14, 18, 19]. Briefly, a sterile disposable swab was attached to the tip of a bovine artificial insemination (AI) gun and wrapped with an external protective plastic sheath. Once inside the uterine cavity, the swab is pushed through a plastic sheath and moved back and forth through the endometrium. Before removal, the swabs were pulled back into the insemination gun to prevent contamination of the cervix and vagina.

#### Reagents and instruments

The universal genomic DNA extraction kit was purchased from Beijing Solarbio Company.

### Methods

#### Ethics approval and consent to participate

We obtained informed consent from the Xiahe County Ranch in the Gannan Tibetan Autonomous Prefecture to use the animals in our study. All animal experiments were conducted in accordance with the relevant guidelines and regulations, in addition to the ARRIVE guidelines, and were approved by the Ethics Committee of Gansu Agricultural University, China (ethics approval file No. GSAU-Eth-VMC-2023-006).

#### Sample DNA extraction

Total DNA was extracted from the samples using a DNA extraction kit purchased from the Beijing Solarbio Company. The extracted DNA was analyzed by 1% agarose gel electrophoresis to detect the microbial genome. All samples were stored at − 20 °C.

#### Macrogenomic testing

The experimental procedure was performed according to the standard protocol provided by Illumina and included sample quality detection, library construction, library quality detection, and library sequencing. Specifically, DNA was fragmented after the genomic DNA of the sample was qualified. The fragmented DNA was subjected to end repair, 3′ end A-tailing, sequencing adapter ligation, adapter purification, library amplification, and library purification to form a sequencing library. After the library passed quality inspection, it was sequenced on the Illumina HiSeq platform with 150 bp paired end reads.

### Data analysis

#### Sequencing data quality control

Raw reads obtained from sequencing contained low-quality sequences. To ensure the quality of the information analysis, the raw reads were filtered to obtain clean reads for subsequent information analysis. The FASTP software filters raw tags and obtains high-quality sequencing data (i.e., clean tags) [20].

#### Macrogenome assembly and gene prediction

1. The software MEGAHIT was used for metagenomic assembly [21], and contig sequences shorter than 300 bp were filtered out.
2. The assembly results were evaluated using QUAST software [22].
3. The Metagene Mark software was used with default parameters to identify the coding regions in the genome [23].

#### Constructing non-redundant gene sets

The MMseqs2software was used to remove redundancy with a similarity threshold set to 95% and a coverage threshold set to 90% [24].

#### Functional gene annotation analysis

1. GO annotation: By comparison with the Pfam database, genes corresponding to Pfam in the GO database were annotated using functional information. The annotation information corresponding to these nodes in the GO database represents the annotation information of the genes in the sequenced genome [25].
2. KEGG annotation: By comparing the non-redundant gene protein sequences with the protein sequences in the KEGG database using BLAST (with an expected value of 1e-5), the most similar sequence in the KEGG database is found. The annotation information of the sequence, the corresponding KO number, and the position in the biological pathway corresponding to the KO number are the annotation information, KO number, and position in the biological pathway of the corresponding gene in the sequenced genome [26].
3. eggNOG annotation: The most similar sequence in the eggNOG database was found by comparing non-redundant gene protein sequences with the eggNOG database using BLAST (with an expected value of 1e-5). The annotation and classification information corresponding to a sequence is the annotation and classification information of the corresponding gene in the sequenced genome [27].
4. CAZy annotation: All families that met the filtering threshold were found using the HMMer software to compare the protein sequences of non-redundant genes with the hidden Markov models of each family in the CAZy database. This method can identify carbohydrate-active enzymes in the genome and analyze the number of conserved carbohydrate-related functional domains that they contain [28].
5. CARD annotation: Using the RGI tool in the CARD database to compare the protein sequences of non-redundant genes with those in the database, the corresponding sequence in the database was found, and the corresponding resistance genes and resistance-related information were obtained [29].

#### Analysis of the similarity of functional genes at different periods of estrus

1. ANOSIM analysis: Based on the KEGG’s KO and eggNOG’s NOG hierarchy, an ANOSIM analysis was conducted using *R*’s vegan package. Box plots were drawn based on the binary Jaccard and Bray–Curtis algorithm grouping distance matrices to test for significant differences between the samples in distinct groups.
2. Functional gene correlation network analysis: Correlation network maps are a type of correlation analysis. Using the eggNOGNOG hierarchy, the top 80 functional genes were selected based on their abundance. Spearman’s correlation analysis (including positive and negative correlations) was conducted and statistically tested based on the abundance and variation of each functional gene in each sample. Data groups with a correlation > 0.5 and a *p*-value < 0.05 were selected, and a correlation network diagram was drawn using R.

#### Species annotation and abundance analysis

The species composition and relative abundance of the samples were obtained according to the species information of the sequences aligned to the NR database.

1. Species composition bar chart: Python was used to draw bar charts of species at the kingdom, phylum, class, order, family, genus, and species levels. The species composition and proportions of varied species in each sample were visually displayed.
2. ANOVA analysis of inter-group differences in species composition: ANOVA was used to analyze the significant differences in species composition among the four groups of samples and to obtain a heatmap of species-level differentially abundant species.
3. Random forest analysis of species: By constructing multiple decision trees, the samples are classified. Feature species that significantly affect the differences between samples were obtained.
4. Correlation analysis of species: The top 80 abundant species (at the level of the phylum to species) were selected based on the abundance and variation of each species in each sample. The Spearman algorithm was used to perform correlation analysis (including positive and negative correlations) and statistical tests. Data groups with correlation coefficients > 0.5 and *p*-values < 0.05 were selected. A correlation network diagram was drawn using R.

## Results and analysis

### Analysis of differences in the number of gene samples from different treatment groups

**Fig. 2:**
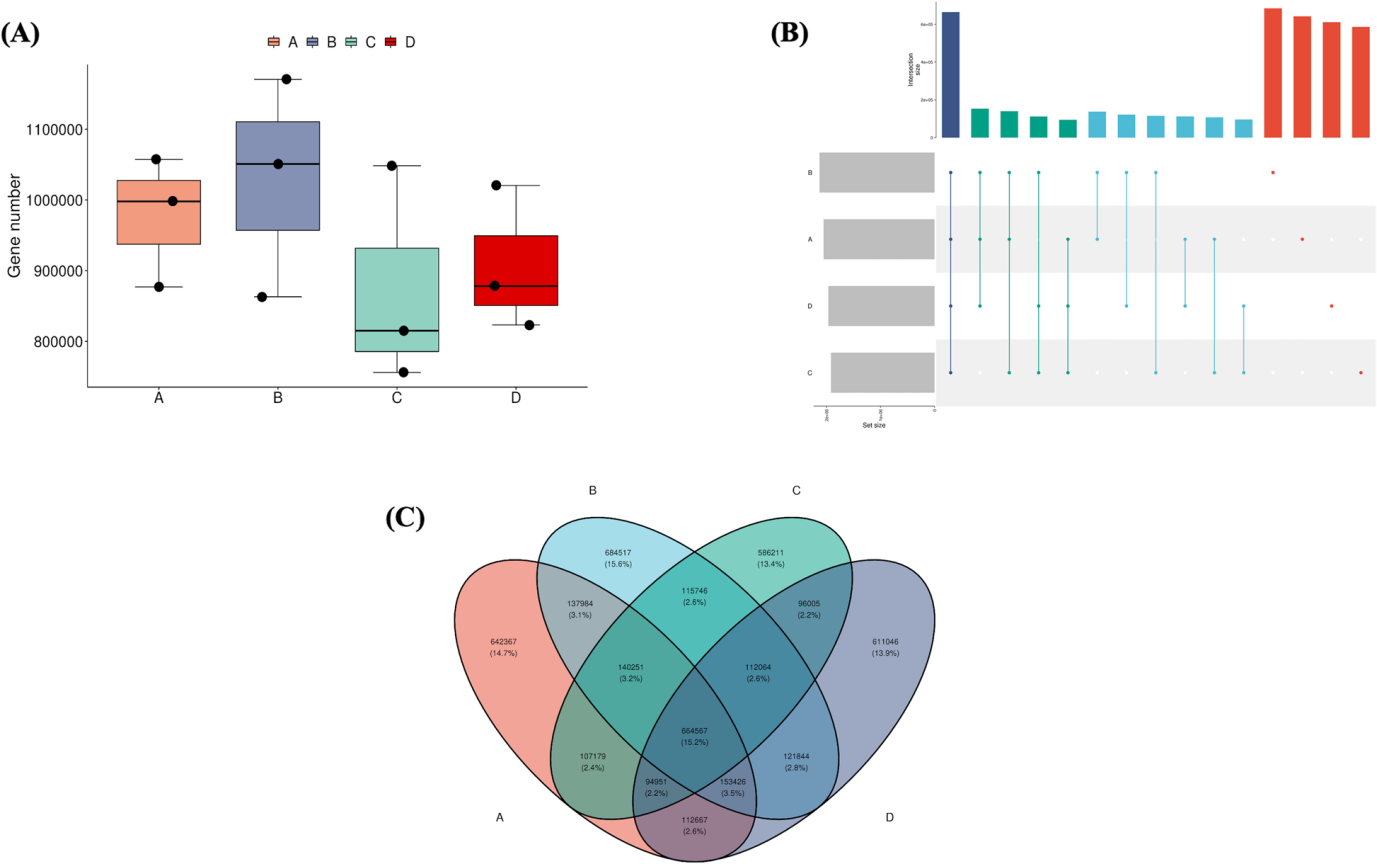
Analysis of differences in the number of genes between groups through mapping. **A** Box plot. **B** Upset diagram (number of subgroups 3 to 9). **C** Venn diagram (number of groups 2 to 5)

After removing redundancy using the MMseqs2 software, 4,408,022 non-redundant genes were obtained. To analyze the differences in gene numbers between the groups, box plots (Fig. 2a), Venn diagrams (Fig. 2b), and Upset (Fig. 2c) plots were drawn (upset plots were drawn between samples/groups 3–9, and Venn diagrams were drawn between samples/groups 2–5). It was found that there were 664,567 non-redundant genes shared among the four estrous cycle periods, accounting for 15.2% of all non-redundant genes between groups. Among these, the estrus period had unique non-redundant genes, totaling 684,517 and accounting for 15.6% of all non-redundant genes between the groups; the number of genes was relatively dispersed and followed a normal distribution. The pre-estrus period contained 642,367 unique non-redundant genes, accounting for 14.7% of all non-redundant genes between the groups; the number of genes was relatively high, followed by a right-skewed distribution. The post-estrus period contained 586,211 unique non-redundant genes, accounting for 13.4% of all non-redundant genes between the groups; the number of genes was relatively dispersed, with a right-skewed distribution. The diestrus period had 611,046 unique non-redundant genes, accounting for 13.9% of all non-redundant genes between groups; the number of genes was relatively high, followed by a left-skewed distribution.

### Annotated analysis of common database functions

#### GO annotation analysis

**Fig. 3:**
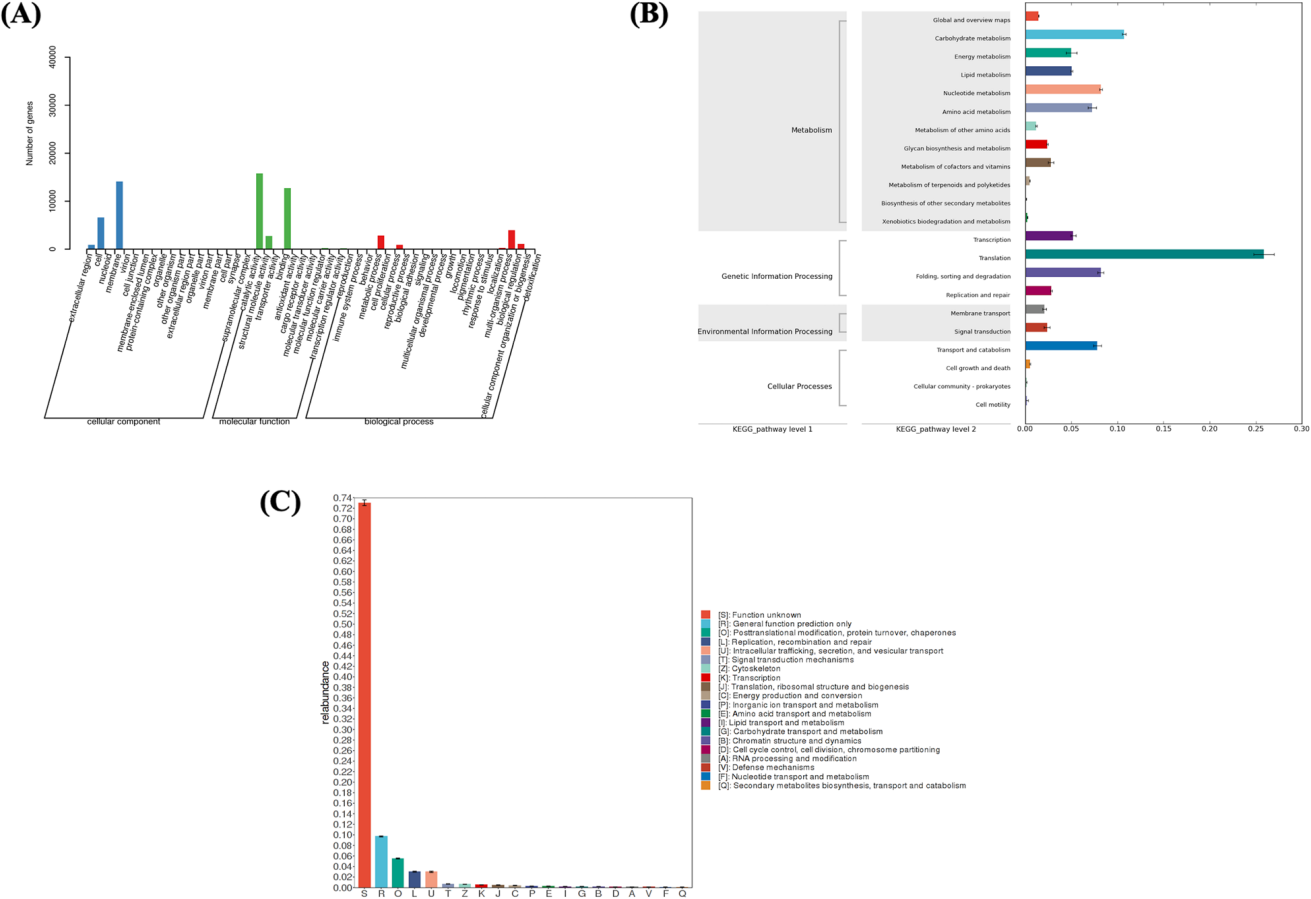
Function of enrichment in the Yak uterus. **A** GO annotation analysis. **B** KEGG annotation analysis. **C** eggNOG annotation analysis

In the GO database, a total of 26,523 genes were annotated. Results showed that the functional genes in the samples were mainly located in the cell membrane structure and intracellular and extracellular matrix; catalytic activity, binding activity, and structural molecular activity were prominent. These genes participate in organism processes, metabolic processes, cellular processes, and biological regulation (Fig. 3a).

#### KEGG annotation analysis

**Fig. 4:**
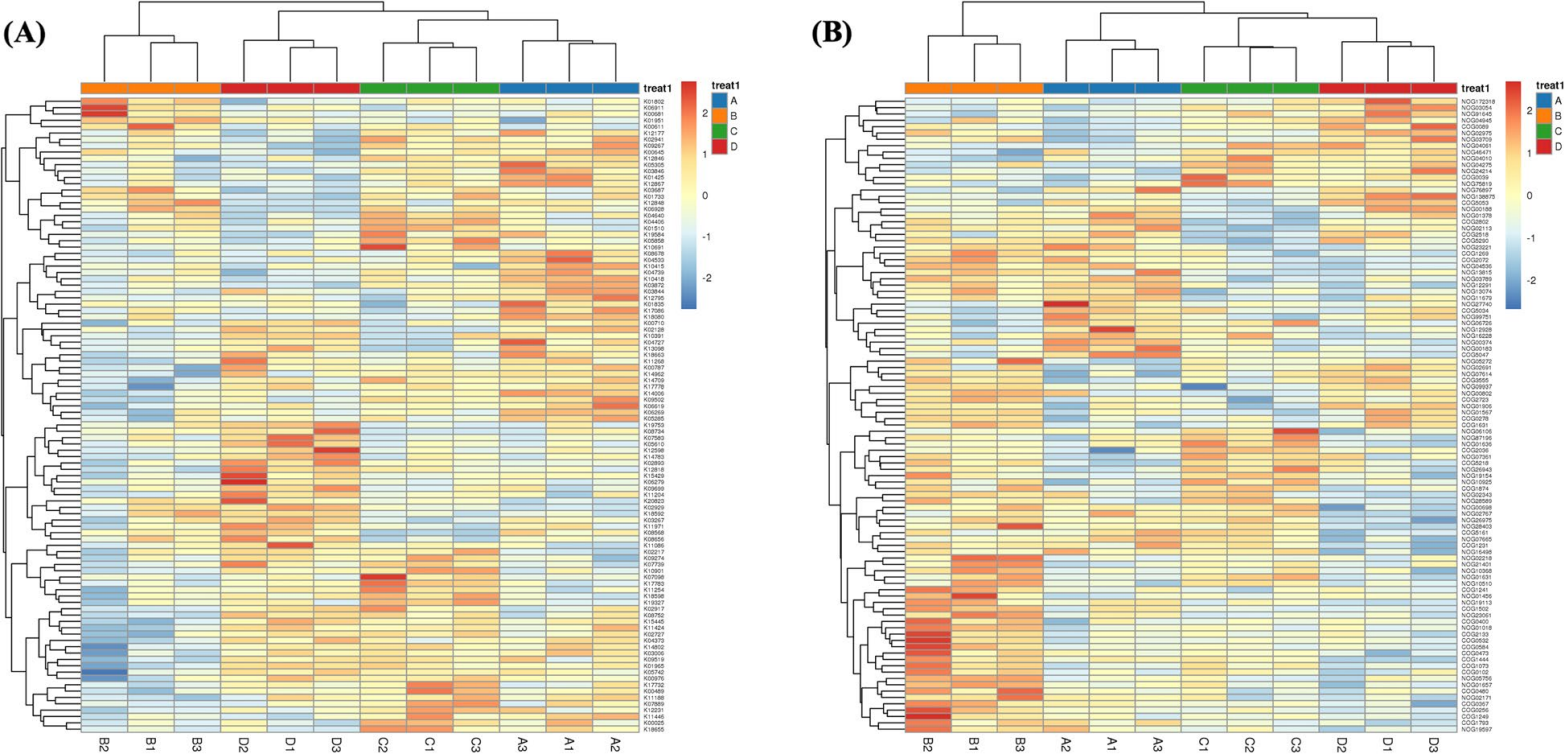
Differential function of enrichment in the different reproductive stages of the yak uterus. **A** KEGG differential function gene abundance heat map. **B** eggNOG differential function gene abundance heat map

The FMAP software was used to annotate the sample genes with KEGG and perform differential module analysis to determine the possible metabolic pathways involved. A total of 20,555 genes were annotated using the Kyoto Encyclopedia of Genes and Genomes (KEGG) database (S1). Results showed that the translation function of the functional genes in the sample was prominent, followed by metabolic activities (such as carbohydrate metabolism, nucleotide metabolism, transport and decomposition metabolism, and amino acid metabolism). Other functions included energy and lipid metabolism (Fig. 3b). According to the KEGG differential functional gene abundance heatmap, alpha-1,2-mannosyltransferase, apoptosis-inducing factor 1, and Abelson tyrosine-protein kinase 1 were significantly upregulated in the pre-estrus period; gamma-glutamyl transpeptidase/glutathione, hydrolase, and ornithine carbamoyl transferase were significantly upregulated in the estrus period; E3 ubiquitin-protein ligase UBR4 and cholesterol 7alpha-monooxygenase were significantly upregulated in the post-estrus period; and ATP-dependent RNA helicase DOB1, SHC-transforming protein 1, and small nuclear ribonucleoprotein B and B′ were significantly upregulated in the inter-estrus period (Fig. 4a). All these annotated KO functions can be found in S3.

#### eggNOG annotation analysis

The eggNOG database annotates 268,572 genes (S2), approximately 73% of which have unknown functions. Around 10% of functional genes can be used for general functional prediction, whereas approximately 5% are involved in post-translational modifications and protein conversion. About 3% of the functional genes are involved in gene replication, recombination, and repair, whereas another 3% are involved in intracellular transport, secretion, and vesicular transport. Other functions include signal transduction and transcription (Fig. 3c). According to the differential functional gene abundance heatmap generated by eggNOG, the Sec-23 interacting protein (sec23) and transport proteins were upregulated during the pre-estrus period; however, their main functions are unknown. During estrus, dihydrolipoyl dehydrogenase, aldose sugar dehydrogenase, translation initiation factor IF-2, 5-methylcytosine-specific restriction enzyme B, RAB GTPase-activating protein, and glycerophosphoryl diester phosphodiesterase are upregulated; their main functions are enriched in carbohydrate and amino acid metabolism. During the post-estrous period, leucine zipper-EF-hand-containing transmembrane proteins that catalyze the reversible oxidation of malate to oxaloacetate (by similarity) are upregulated; their main functions are enriched in intracellular transport and secretion. During the diestrus period, the integrase core domain and TatD DNase family proteins were upregulated; their main functions are enriched in post-translational modification of proteins (Fig. 4b). All these annotated eggNOG class can be found in S4.

### Primary focused physiological functions

#### CAZy annotation analysis

**Fig. 5:**
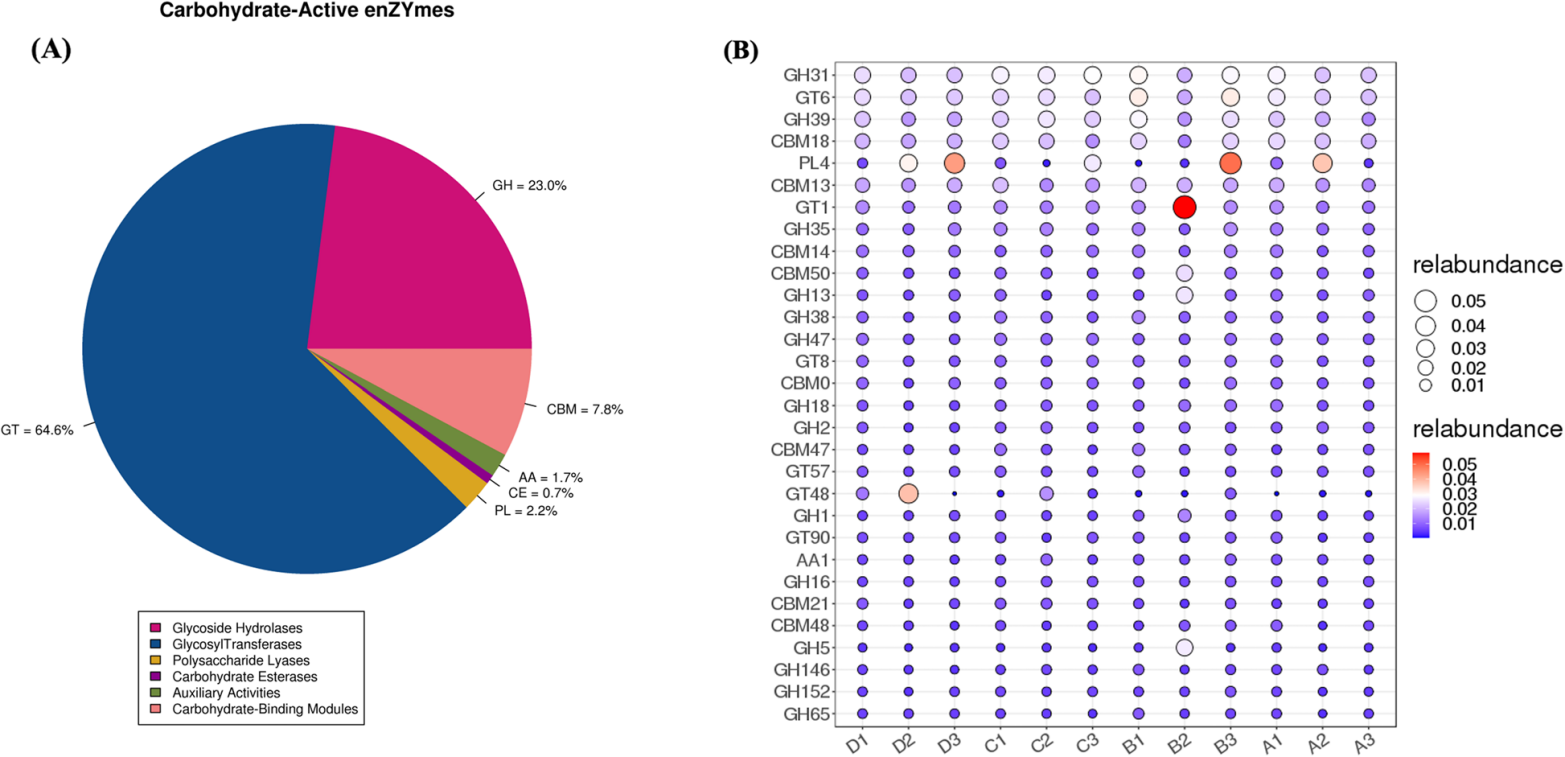
Function of enrichment in the Yak uterus. **A** CAZy annotation analysis. **B** Carbohydrate Enzyme Content Chart

The CAZy database contains annotated 7924 genes, with the highest number belonging to GT (5118) and accounting for 64.6% of all carbohydrate-active enzymes. This is followed by GH (1882) and CBM (618), accounting for 23.0 and 7.8% of all carbohydrate-active enzymes, respectively (Fig. 5a). By counting the number of annotated families (enzyme families) in each sample (Fig. 5b), we found that PL4 had the highest relative abundance in the pre-estrus phase, and that GT1 had the highest relative abundance in the estrus phase, followed by PL4. GH31 had the highest relative abundance in the post-estrus phase, and PL4 had the highest relative abundance in the inter-estrus phase, followed by GT48.

#### CARD resistance gene annotation analysis

**Fig. 6:**
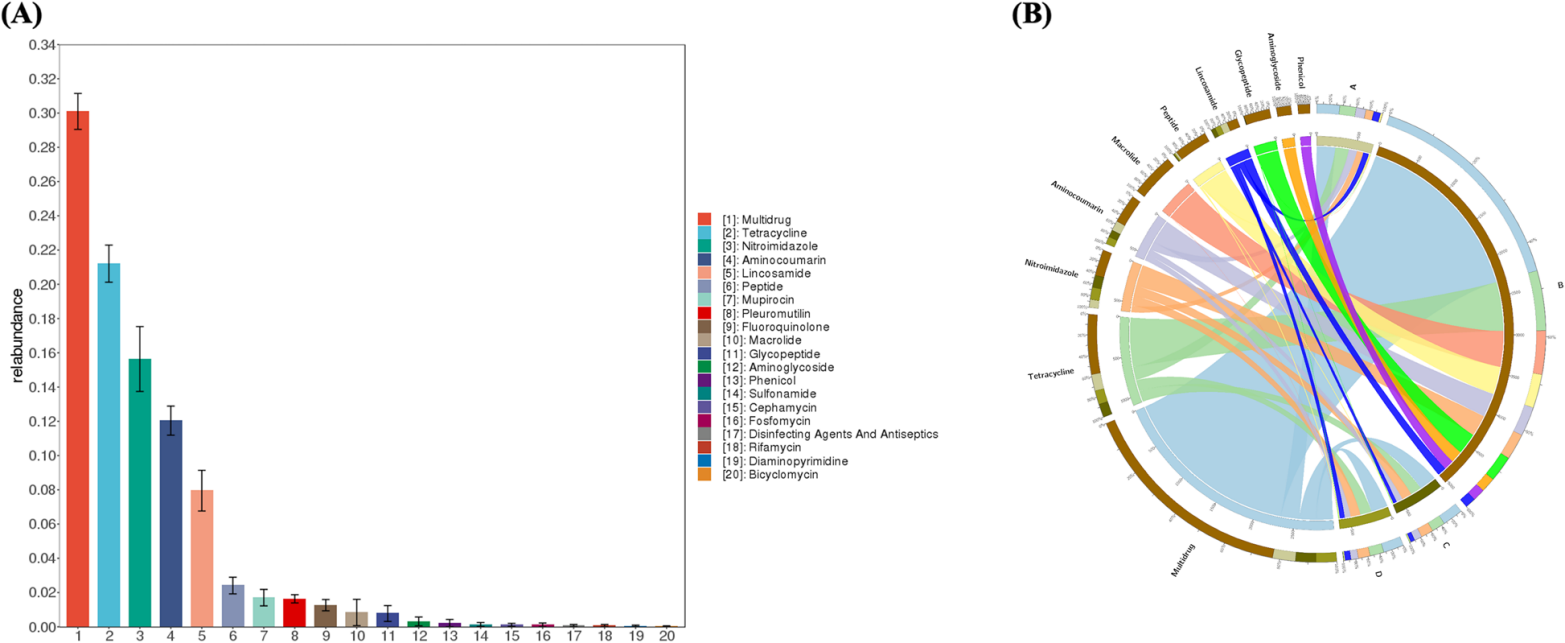
Function of enrichment in the Yak uterus. **A** Antibiotic resistance gene abundance statistics. **B** CARD antibiotic resistance gene circle map

A total of 597 genes resistant to 20 common antibiotics were annotated in the CARD antibiotic resistance gene database (Fig. 6a), including 179 multidrug-resistant genes (30%), 125 tetracycline-resistant genes (21%), 92 nitroimidazole-resistant genes (15.5%), 71 aminoglycoside-resistant genes (12%), and 47 lincomycin-resistant genes (8%). This indicates that yak uteri contain a highly diverse array of antibiotic resistance genes. The circular composition map of the CARD antibiotic resistance genes showed that the uteri of yaks in estrus included a variety of antibiotic resistance genes, far exceeding other periods; the three most abundant resistance genes were multidrug-resistant, tetracycline-resistant, and midecamycin-resistant genes (Fig. 6b).

### Similarity analysis of functional genes in different estrus periods

#### ANOSIM analysis

**Fig. 7:**
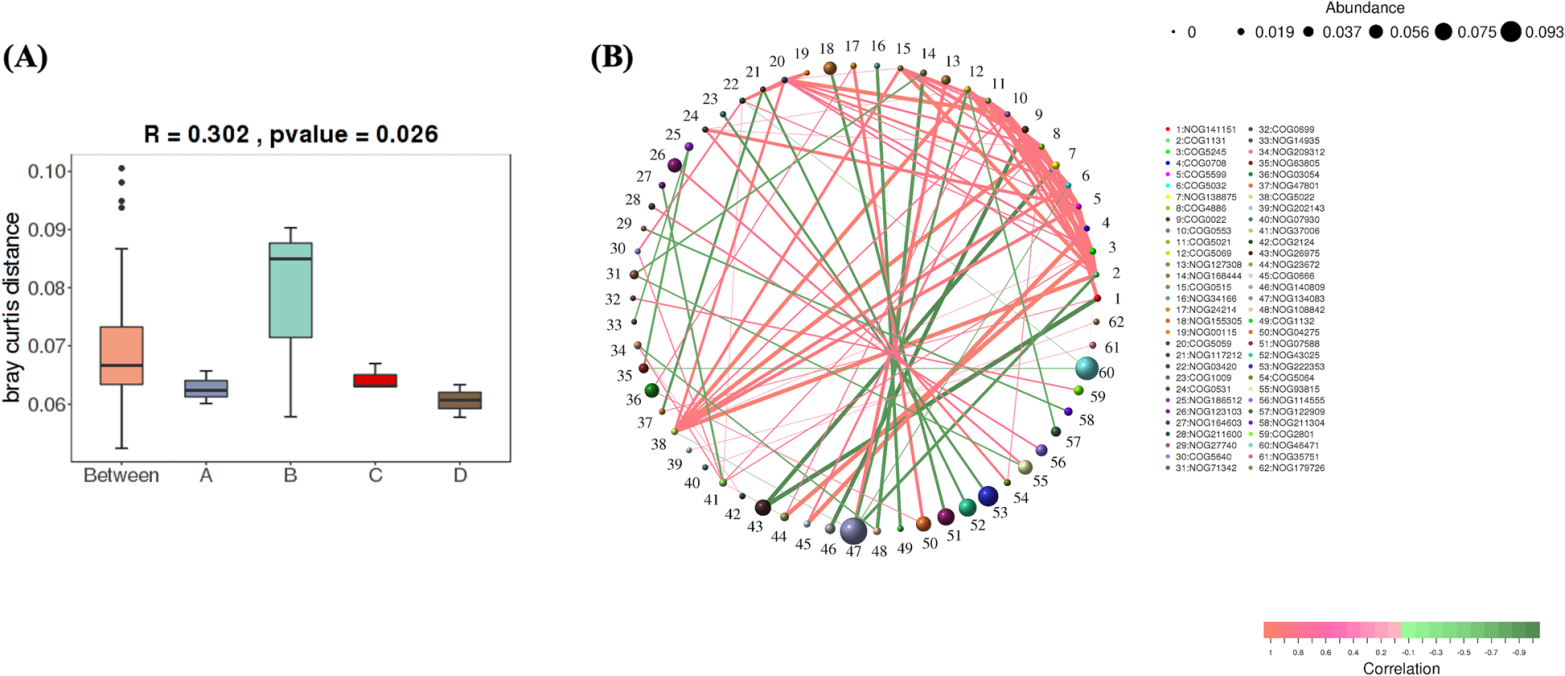
Analysis of the similarity of functional genes at different reproductive periods. **A** Anosim plots: inter-sample distances for functional genes. **B** Functional gene correlation network map

As shown in Fig. 7a, there are significant differences in the functional genes between the estrus group and the other three groups. There were also differences in the functional gene distribution among the three groups of yaks with different estrus cycles. ‘Between’ represents the difference between groups, whereas the others are within groups. The distance within a group is smaller than that between groups, which indicates that the grouping is effective (Fig. 7a).

#### Correlation network analysis of functional genes

Results showed that endonuclease and reverse transcriptase were the most dominant genera in the sample and were negatively correlated. The functional gene represented by the number 38 (endonuclease) was positively associated with almost all other functional genes. The functional gene represented by the number 47 (endonuclease) was mutually constrained to 12 (microtubule-associated monooxygenase, calmodulin, and LIM domain-containing protein) and 2 (ABC transporter) other functional genes (Fig. 7b). All these annotated eggNOG class can be found in S5.

### Analysis of microbial species composition

#### Species composition genus level distribution map

**Fig. 8:**
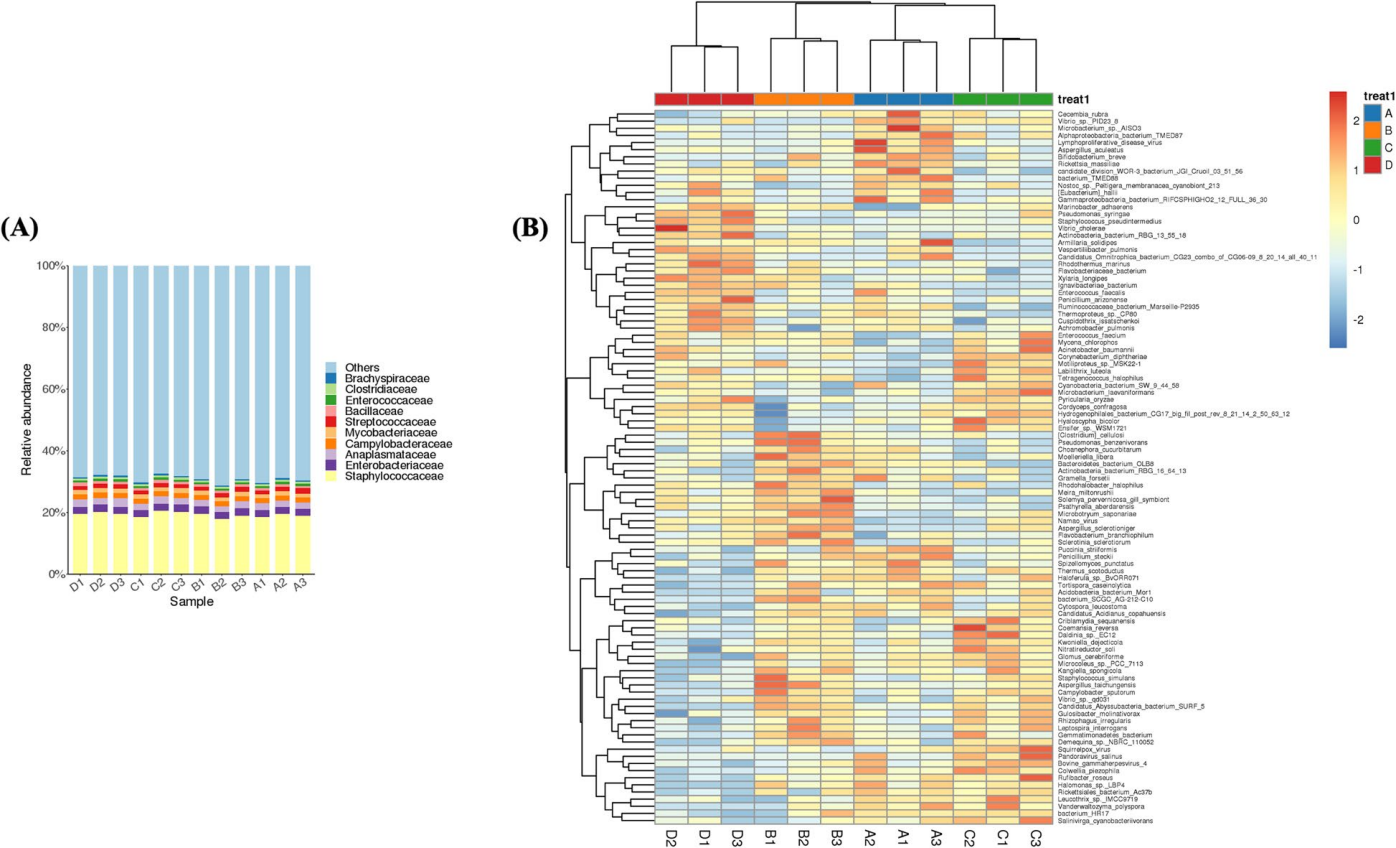
Composition of species and differences. **A** Species composition distribution map. **B** Differential species abundance heat map

Results showed that the dominant species in the four periods were *Staphylococcus aureus*, *Mycoplasma phagocytophilum*, *Klebsiella pneumoniae*, and *Escherichia coli* (Fig. 8a).

#### Heat map of species-level abundance of species differing between groups

Results showed that the dominant species in the pre-estrus period were *Micrococcus* and *Proteus*. In contrast, the dominant species during the estrus period were *Pseudomonas, Clostridium, Flavobacterium, Bacillus,* and *Staphylococcus*. The dominant species in the late estrus period were *Acinetobacter, Bacillus,* and *Proteus*, while the dominant species in the diestrus period were *Pseudomonas*, *E. coli*, and *Bacillus* (Fig. 8b, Table 1).

**Table 1:** Microbes detected in different estrous cycles

*Stage*	*Microbes*
*pre-estrus* *estrus* *late estrus* *diestrus*	Micrococcus and Proteus Pseudomonas, Clostridium, Flavobacterium, Bacillus, and Staphylococcus Acinetobacter, Bacillus, and Proteus Pseudomonas, *E. coli*, and Bacillus

#### Species random forest analysis

**Fig. 9:**
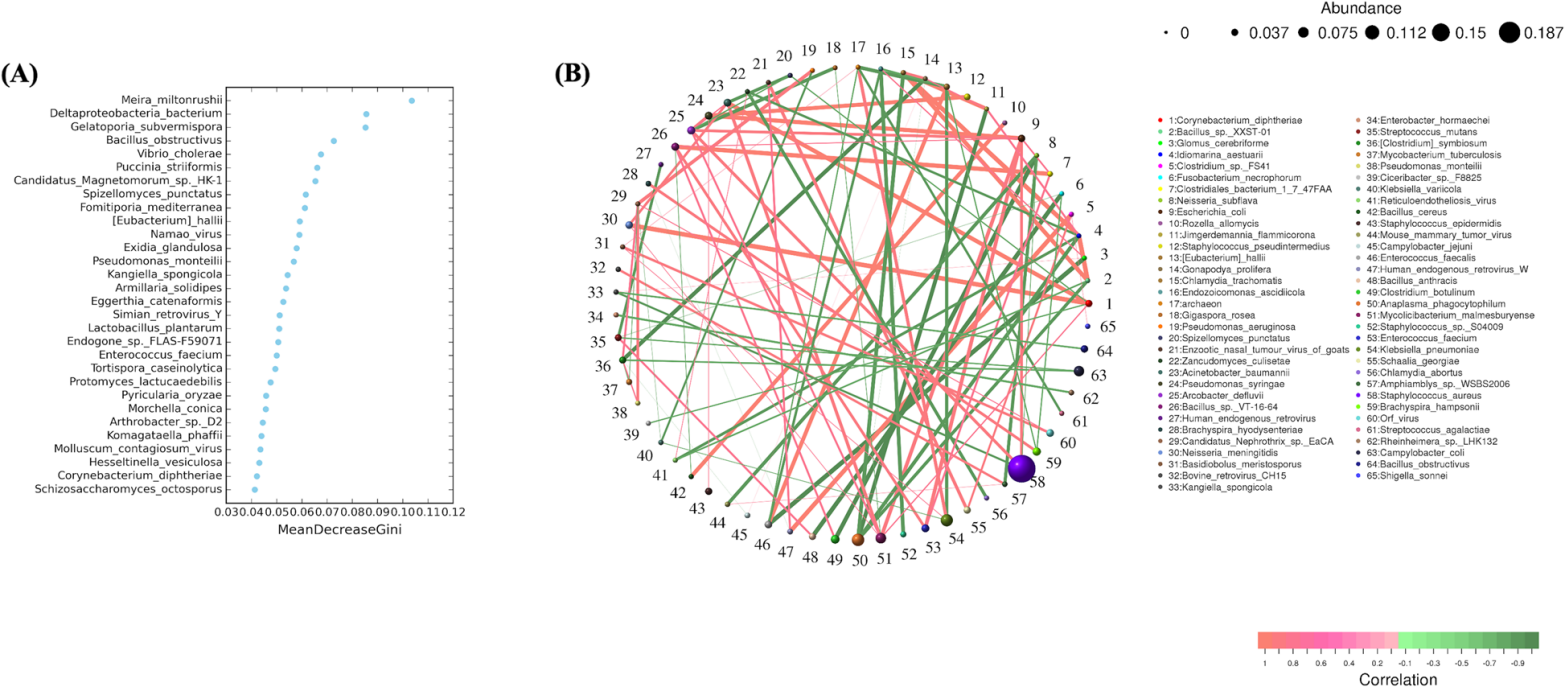
Composition of species and differences. **A** Species composition distribution map. **B** Differential species abundance heat map

The results of the species importance ranking showed that *Meira miltonrushii*, *Deltaproteobacteria bacterium*, *Gelatoporia subvermispora*, and *Bacillus obstructive* were the feature species that had a significant impact on the differences between samples (Fig. 9a).

#### Species correlation analysis

Species correlation analysis showed that *Staph.aureus* was the most dominant genus in the network, with positive correlations with *E. coli* and *A. defluvii****.*** *Anaplasma phagocytophilum* was negatively correlated with *Chlamydia trachomatis*, *Jimgerdemannia flammicorona*, *ldiomarina aestuarii*, and *Staphylococcus pseudintermedius* (Fig. 9b).

## Discussion

The uterus plays a crucial role in maintaining pregnancy in yaks. Previous studies have mainly focused on the uterine muscular arterioles [30], hypoxia-inducible factor-1α (HIF-1α) [15], DNA damage-inducible transcript 3 (DDIT3) [31], follicle-stimulating hormone receptor (FSHR) [32], and estrogen receptor genes (ER) [33]. However, little is known about the diversity of uterine microbiota and its impact on reproductive health in different reproductive states. This study utilized metagenomics to investigate the microbial characteristics of the yak uterus during different reproductive stages, thereby providing a new perspective for studying the physiological regulation and mechanisms of reproductive diseases in yaks. This approach offers a promising avenue for understanding the role of the microbiota in regulating reproductive processes and preventing reproductive tract-related disorders.

One thousand two hundred ninety-three microbial genera, consisting of 3401 species, were identified from the samples. Among them were the most common bacteria found in the endometrium, such as *Acinetobacter*, *Pseudomonas*, *Bacillus*, and *E. coli*; *Lactobacillus* was not the dominant species. The bacterial communities in the endometrium differ significantly from those in the vagina [5]. Studies have shown that *Acinetobacter* and *Pseudomonas* can promote the utilization of non-fibrous carbohydrates, which further improves the immune, digestive, and developmental functions of the bovine gastrointestinal tract [34]. Therefore, microbiota may participate in the immune response of the animal uterus. However, *P. aeruginosa* is known to cause multiple drug resistance and can increase tumor necrosis factor levels in the serum of infected mice, exacerbating colonic and systemic pro-inflammatory responses [35]. This indicates that the prevention of endometritis and other diseases is crucial. Additionally, patients with colorectal cancer have an increased abundance of *baiP* and *baiJ* in *B. subtilis* compared to healthy individuals [36]. Atypical enteropathogenic *E. coli* can also disrupt the stability of immune microbiota and cause ulcerative colitis [37]. Therefore, exploring the diversity of the uterine microbiota in animals in different reproductive stages is important for analyzing the correlation between the microbiota and reproductive regulation.

Comparison of the vaginal microbiota among the four reproductive periods revealed variations in species abundance and diversity. During the pre-estrus period, *Micrococcus* and *Proteus* were the dominant species, while *Pseudomonas*, *Clostridium*, *Flavobacterium*, *Bacteroides* and *Staphylococcus* dominated during the estrus period. In the late estrus period, the dominant species were *Acinetobacter*, *Bacillus*, and *Proteus*; during the diestrus period, *Pseudomonas*, *Escherichia coli*, and *Bacteroides* were dominant. The abundances of *Pseudomonas*, *Proteus*, *Cellulomonas*, and *Hemophilus* remained low during the three periods, except during estrus, where their abundances significantly increased. Bacterial diversity differed significantly between the luteal and follicular phases of the Indian buffalo reproductive cycle. During the luteal phase, *Micrococcus* was more abundant, whereas *Massilia* and *Methylobacter* were more prevalent during the follicular phase [38].

These results are similar to those obtained in the present study, indicating that changes in the microbial community are closely related to changes in the reproductive status of animals. Moreover, analysis of the fecal microbiota showed that *Clostridium* and *Bacteroides* were the characteristic intestinal microbiota of water buffaloes during estrus, with only *Bacteroidetes* present during this period [39]. Similarly, *Tenericutes*, *Firmicutes*, and *Bacteroidetes* were the most abundant vaginal microbes in cows during estrus [12]. In the vagina of giant pandas, *Bacteroides* were maintained at low levels, but their abundance significantly increased during estrus [40]. Therefore, these results suggest that *Bacteroides* may serve as a marker of estrus in yaks.

Pathogenic bacteria (such as *Clostridium*, *Pseudomonas*, *Proteus*, and *Actinomyces*) were also detected in the samples. In cows with uterine inflammation, the relative abundances of *Bacteroides* and *Clostridium* was higher, whereas those of *Proteobacteria* and *Tenericutes* was lower. Thus, an increase in the abundances of *Pseudomonas* and *Clostridium* was strongly associated with uterine inflammation [41]. Similarly, endometritis in sows has been linked to an increased relative abundance of *Actinobacillus*, *Streptococcus*, *Clostridium*, *Porphyromonas*, and *Bacteroides* [42]. These findings suggest that changes in the uterine microbiota, which significantly increases the number of pathogenic bacteria, may induce uterine diseases. The uteri of women and animals with uterine diseases show an increased abundance of *Proteobacteria* (such as *E. coli* and *Enterococcus*), *Bacteroides* (such as *Prevotella*), and *Actinomyces* (such as *Gardnerella*) [43]. Moreover, women with endometriosis have increased numbers of rod-shaped bacteria in their uteri, including *Enterobacteriaceae*, *Flavobacterium, Pseudomonas*, and *Streptococcus* [44]. These findings indicate that changes in the diversity of uterine microbiota are closely related to the occurrence of reproductive diseases. Changing the uterine microbiota may provide new strategies for treating uterine diseases in animals.

Through annotation of the CARD resistance gene database, it was discovered that the uteri of yaks at different reproductive stages contained numerous antibiotic resistance genes. The number and types of these genes increased significantly during estrus, surpassing other periods by a large margin. The three most prevalent resistance genes during estrus are multidrug, tetracycline, and macrolide resistance genes. Multidrug resistance (MDR) may be induced by various mechanisms linked to a complicated process of multiple genes, factors, pathways, and steps [45], such as elevated xenobiotic metabolism, growth factors, increased DNA repair capacity, and genetic factors [46]. Tetracycline resistance has been documented to be highly prevalent [47]. The main mechanisms involved are ribosomal protection, efflux pumps, drug target modification and enzymatic alteration [48]. Alteration at the ribosomal level has been shown to play a more important role in tetracycline resistance than the efflux systems [47]. The mechanisms of macrolide resistance have been extensively studied. In addition to erm-mediated rRNA methylation and mef-mediated efflux [49], emphasis has been placed on mutations in *23S rRNA* and ribosomal proteins [50]. These findings suggest that antibiotic use should be strictly regulated during estrus in animals. Galvao et al. [41] demonstrated that bacterial tolerance is crucial for maintaining uterine health. Moreover, de Oliveira et al. [51] identified that ceftriaxone could enhance the recovery rate of bovine endometritis and increase milk yield and fertility for 300 days after calving. Therefore, judicious use of antibiotics can treat and prevent reproductive diseases caused by bacterial infections in animals, while aiding in the development of resistance.

In summary, the diversity of microorganisms in yak uteri is associated with different reproductive states. Additionally, microorganisms predominantly regulate animal reproduction through pathways such as carbohydrate decomposition, amino acid metabolism, and drug resistance. This study utilized metagenomic technology for the first time to investigate the microbial characteristics of yak uterine microbiota across various reproductive stages, providing a reference for exploring the physiological regulatory functions and mechanisms related to reproductive diseases in yaks.

### Supplementary Information

**Additional file 1.**

## Data Availability

The datasets generated and analysed during the current study are available in the Sequence Read Archive (SRA) repository, https://www.ncbi.nlm.nih.gov/bioproject/PRJNA974397/.

## Abbreviations

CS: Cotton swab
LBR: Live birth rate
FSHR: Follicle-stimulating hormone receptor
E1: Estrone
E2: Estradiol
LH: Luteinizing hormone
FSH: Follicle-stimulating hormone
IVF: In vitro fertilization
RIF: Repeated implantation failure
E3: Estriol
MDR: Multidrug resistance
*E. coli*: *Escherichia coli*
*P. aeruginosa*: *Pseudomonas aeruginosa*
*Staph.aureus*: *Staphylococcus*
*A. defluvii*: *Arcobacter defluvii*
